# Le traitement du décollement de rétine du pseudophaque: vitrectomie sans indentation épisclérale VERSUS Chirurgie Ab externo

**DOI:** 10.11604/pamj.2019.32.44.15489

**Published:** 2019-01-24

**Authors:** Ahmed Ghazza, Maha Bakhsh, Ibtissam Hajji, Abdeljalil Moutaouaki

**Affiliations:** 1 Service d’Ophtalmologie, CHU Mohamed VI, Marrakech, Maroc

**Keywords:** Décollement de rétine, pseudophaquie, vitrectomie, indentation épisclérale, Retinal detachment, pseudophakic, vitrectomy, scleral buckle

## Abstract

Plusieurs facteurs prédisposent à la survenue d'un décollement de rétine rhegmatogène, notamment la chirurgie de cataracte, la myopie, les lésions dégénératives de la périphérie rétinienne ainsi que la notion de traumatisme oculaire. L'objectif de notre étude est de comparer les résultats anatomiques et fonctionnels des deux techniques chirurgicales (Ab interno Vs Ab externo) au sein de notre structure. Il s'agit d'une étude descriptive rétrospective, réalisée au sein du service d'ophtalmologie du CHU Mohamed VI de Marrakech, étalée sur 3 ans, allant de janvier 2013 à décembre 2015, comparant deux groupes d'étude , le premier groupe opéré par voie externe (Cryo application des déhiscences avec une indentation épi sclérale) et le 2^ème^ opéré par voie endo-oculaire. Le groupe A ou groupe ab externo était constitué de 26 yeux (26 patients) contre 22 yeux (22 patients) dans le groupe B ou groupe vitrectomie avec tamponnement interne. Dans le groupe ab externo, l'âge moyen était de 54,92 ans contre 51,64 ans dans le groupe ab interno, avec une légère prédominance masculine dans les 2 groupes. La réapplication rétinienne après la 1^ère^ intervention était obtenue chez les deux groupes sans différence significative, aux alentours de 80,76% chez les patients du groupe A versus 81,82% du deuxième groupe. Les causes d'échec dans les deux groupes étaient dues à la présence d'une prolifération vitréorétinienne avancée (4 cas), d'une déchirure de novo (3 cas) et de déchirures non vues à l'examen initial (2 cas). Tous ces cas ont été repris par un abord ab interno, consistant chez les patients du groupe A en une vitrectomie complète avec révision de l'indentation +/- pelage de membrane limitante interne avec tamponnement interne par gaz et chez les patients du groupe B en un complément de vitrectomie avec dissection de la PVR et un tamponnement interne par huile de silicone. Après un recul moyen de 12 mois, l'acuité visuelle finale de nos patients était sans différence significative entre les 2 groupes, avec plus que le tiers des patients avaient retrouvé une acuité visuelle entre 1/10 et 5/10, aux alentours de 34,61% au niveau du groupe ab externo, et 36,36% pour le groupe ab interno. Certes, avec les avancées technologiques en terme de vitrectomie, la tendance actuelle est la chirurgie endo-oculaire, cependant la chirurgie ab-externo garde sa place principalement lors des DR avec des déchirures visibles, bien accessibles à l'indentation sans prolifération vitréorétinienne avancée (PVR A-Bà).

## Abstract (en)

Several factors predispose to the occurrence of rhegmatogenous retinal detachment, including cataract surgery, myopia, and degenerative lesions of the peripheral retina as well as eye trauma. This study aims to compare the anatomical and functional outcome of the two surgical techniques (AB interno vs AB externo) in our Hospital. We conducted a descriptive, retrospective study in the Department of Ophthalmology at the University Hospital Mohamed VI in Marrakech, over a period of 3 years, ranging from January 2013 to December 2015. During the study period we compared two groups of people: the first group undergoing surgery by an external approach (cryoapplication of the dehiscences with episcleral indentation) while the second undergoing surgery by endo-ocular approach. Group A or AB externo group included 26 eyes (26 patients) while Group B or vitrectomy with internal tamponade group included 22 eyes (22 patients). In the AB externo group, the average age was 54.92 years against 51.64 years in the AB interno group, with a slight predominance of males in both groups. After the first surgery, retinal reapplication was obtained in both groups with no significant difference, (about 80.76% in Group A versus 81.82% of the second group). In both groups failure was caused advanced vitreoretinal proliferation (4 cases), de novo breaks (3 cases) and breaks which had not been detected on first examinations (2 cases). All these cases underwent reoperation by AB interno approach: patients of group A underwent complete vitrectomy with revision of the indentation +/- internal limiting membrane peeling with internal gas tamponade while patients of group B underwent complementary vitrectomy with dissection of proliferative vitreoretinopathy (PVR) and an internal tamponade with silicone oil. After a mean follow-up period of 12 months, there was no significant difference in visual acuity between the 2 groups, with more than one third of the patients who had regained visual acuity between 1/10 and 5/10 (about 34,61% in AB externo group and 36,36% in AB interno group). Given the technological advances in vitrectomy, the current trend is the endo-ocular surgery however ab-externo surgery is mainly used in the treatment of retinal detachment with visible breaks which can be easily treated with indentation without advanced vitreoretineal proliferation (PVR A-B).

## Introduction

La chirurgie rétinienne a considérablement évolué ces vingt dernières années avec le développement de la chirurgie endo-oculaire, de même que les connaissances de la physio pathogénie des maladies rétiniennes, impliquant de plus en plus le vitré dans leur genèse et leur évolution. Plusieurs facteurs prédisposent à la survenue d'un décollement de rétine rhegmatogène, notamment la chirurgie de cataracte, la myopie, les lésions dégénératives de la périphérie rétinienne ainsi que la notion de traumatisme oculaire [1]. Bien que les résultats de la chirurgie de cataracte aient beaucoup progressé après l'amélioration des techniques de phaco-exérèse, le décollement de rétine rhegmatogène (DRR) demeure l'une des complications post opératoires les plus sévères. Il survient dans 0,5 à 1,5% des cas après un an de la chirurgie de cataracte, et à peu près 30 à 40% des patients ayant un DR rhegmatogène sont pseudophaques [1]. Le traitement par voie externe associant la rétinopexie et l'indentation épisclérale reste la technique de référence dans la prise en charge chirurgicale du décollement de rétine (DR) du pseudophaque sans prolifération vitréo-rétinienne (PVR) marquée. Cependant avec les récentes avancés dans les techniques et matériaux de vitrectomie, plusieurs chirurgiens ont opté pour l'abord ab-interno comme premier choix plus particulièrement chez les patients pseudophaques. Le but du travail est de comparer les résultats anatomiques et fonctionnels des deux techniques chirurgicales (Ab interno Vs Ab externo) au sein de notre structure.

## Méthodes

Il s'agit d'une étude descriptive rétrospective, réalisée au sein du Service d'Ophtalmologie du CHU Mohamed VI de Marrakech, étalée sur 3ans, allant de janvier 2013 à décembre 2015, comparant deux groupes d'étude, le premier groupe opéré par voie externe et le 2ème opéré par voie interne. Seuls les patients qui auraient pu bénéficier des 2 techniques chirurgicales ont été retenus. Les critères d'exclusion de l'étude comportaient les patients ayant des troubles des milieux (hémorragie intra vitréenne, hyalite..), une récidive de décollement de rétine, une prolifération vitréo-rétinienne initiale stade C ou des déchirures géantes. Nous avons recueilli à partir des dossiers médicaux de nos patients, les données démographiques notamment l'âge et le sexe, la durée de la symptomatologie, l'intervalle entre la chirurgie de la cataracte et la survenue du décollement de rétine, ainsi que l'existence d'une rupture capsulaire ou la réalisation d'une capsulotomie au laser YAG, l'acuité visuelle initiale et enfin les caractéristiques du décollement de rétine en précisant son étendue, l'état maculaire, la présence ou non d'une ou de plusieurs déhiscences et de la prolifération vitréo rétinienne. Les patients du groupe ab externo ont bénéficié d'une Cryo application des déhiscences avec une indentation épisclérale. La réalisation d'un drainage du liquide sous rétinien et ou l'injection d'air ou de gaz dans la cavité vitréenne dépendait du contexte clinique. Concernant les patients du groupe endos-oculaire, ils ont bénéficié d'une vitrectomie centrale et périphérique avec drainage du liquide sous-rétinien et utilisation peropératoire de perfluorocarbone liquide ou air, associée à une endophotocoagulation des déhiscences et un tamponnement interne par gaz. Les variables post-opératoires analysés comportaient le taux de succès anatomique, les causes d'échec anatomique et l'acuité visuelle finale.

## Résultats

Le groupe A ou groupe ab externo était constitué de 26 yeux (26 patients) contre 22 yeux (22 patients) dans le groupe B ou groupe vitrectomie avec tamponnement interne. Dans le groupe ab externo, l'âge moyen était de 54,92ans contre 51,64ans dans le groupe ab interno, avec une légère prédominance masculine dans les 2 groupes. Le délai entre la chirurgie de cataracte et la survenue du décollement de rétine était comparable dans les deux groupes (p = 0,91), ainsi que le délai de consultation (p=0,92). L'acuité visuelle initiale était détériorée chez la majorité des patients des deux groupes, inférieure à 1/10 dans 83,34% des cas du groupe A versus 87,9% des patients du groupe ab interno. La présence d'une rupture capsulaire était notée dans 40,23% des cas dans le groupe ab externo et 37,5% dans le deuxième groupe. L'étendue du décollement de rétine était plus importante dans le groupe ab interno, englobant les 3 quadrants dans 72,82 % alors que dans le premier groupe, le DR ne dépassait pas les 3 quadrants que dans 26,86%, sans différence significative de l'état maculaire entre les deux groupes qui était soulevé dans 84,61 dans le groupe A et dans 86,37% dans le groupe B Les déchirures étaient retrouvées chez tous les patients du groupe ab externo, sans déhiscences visibles dans 27,27% des cas du groupe endo oculaire. Les données cliniques sont résumées dans le Tableau 1.

**Tableau 1:** tableau résumant les différentes données cliniques des 2 groupes

Données cliniques	Groupe A(n=26 (%))	Groupe B(n=22 (%))	p
Intervalle phacoexérèse – DR (mois)	32,75	34,82	0,91
Age du DR (jours)	42,17	39,27	0,92
**Acuité visuelle initiale**			
< 0.1	83,34	87,9	0,45
0.1-0.5	12,5	7,56	0,34
>0.5	4,16	4,54	0,95
Rupture capsulaire	40,23	37,5	0,65
**Etendue du DR**			
1 quadrant	34,67	9	0,0
2-3 quadrants	38,46	18,18	0,24
>3 quadrants	26,86	72,82	0,0007
**Etat de la macula**			
On	15,38	13,63	0,008
Off	84,61	86,37	0,006
**Déchirures**			
0	0	27,27	0,00061
Unique	42,30	31,81	0,19
Multiples	57,71	40,91	0,11

**Figure 1:**
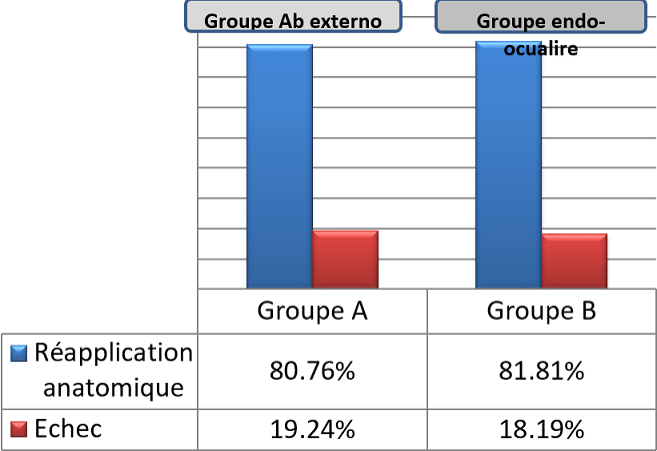
taux de réapplication rétinienne après la première intervention

**Figure 2:**
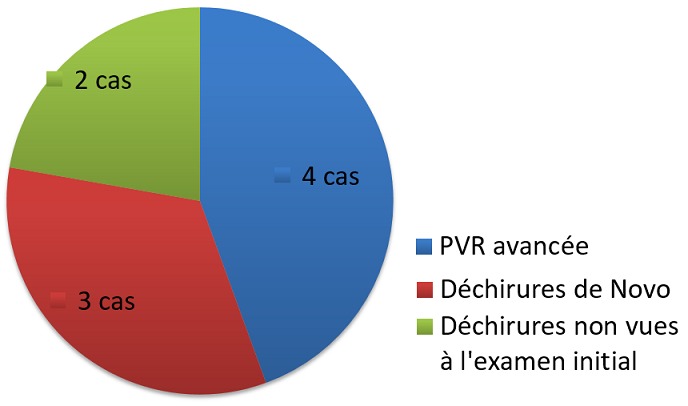
causes d’échec dans les deux groupes d’étude

**Figure 3:**
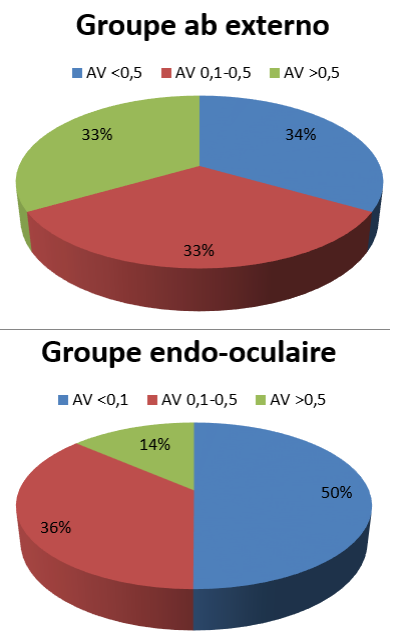
acuité visuelle finale après un recul de douze mois (en pourcentage %)

Le geste chirurgical chez les patients du groupe ab externo a consisté en une indentation épisclérale à l'aide d'une bande de silicone (6,5 mm) avec fixation à l'aide de fil de mersuture non résorbable 5/0. Les patients du deuxième groupe ont bénéficié d'une vitrectomie centrale et périphérique avec drainage du liquide sous-rétinien et utilisation peropératoire de perfluorocarbone liquide ou air, associée à une endophotocoagulation des déhiscences et un tamponnement interne par gaz La réapplication rétinienne après la 1ère intervention était obtenue chez les deux groupes sans différence significative, aux alentours de 80,76% chez les patients du groupe A versus 81,82% du deuxième groupe (Figure 1). Les causes d'échec dans les deux groupes étaient dues à la présence d'une prolifération vitréorétinienne avancée (4 cas), d'une déchirure de novo (3 cas), et de déchirures non vues à l'examen initial (2 cas) (Figure 2). Tous ces cas ont été repris par un abord ab interno ,consistant chez les patients du groupe A en une vitrectomie complète avec révision de l'indentation +/- pelage de membrane limitante interne avec tamponnement interne par gaz et chez les patients du groupe B en un complément de vitrectomie avec dissection de la PVR et un tamponnement interne par huile de silicone. Après un recul moyen de 12 mois, l'acuité visuelle finale de nos patients était sans différence significative entre les 2 groupes, avec plus que le tiers des patients avaient retrouvé une acuité visuelle entre 1/10 et 5/10, aux alentours de 34,61% au niveau du groupe ab externo, et 36,36% pour le groupe ab interno (Figure 3).

## Discussion

L'incidence des DRR a connu une hausse au cours de ces dernières années, ceci est dû en partie à l'augmentation des DRR du pseudophaque [2, 3]. En effet le risque de développer un DRR après chirurgie de cataracte que ça soit par extraction extra capsulaire ou par phacoémulsification peut atteindre 3.6% d'autant plus s'il y a rupture capsulaire postérieure avec issue de vitré ou en cas de capsulotomie au laser YAG, contre 0.005% and 0.0179% chez la population générale [4]. En effet , l'extraction du cristallin et son remplacement par une lentille artificielle plus fine entraine une expansion de la cavité vitréenne et son déplacement en antérieur [5], en plus cette extraction perturbe la fonction de barrière du cristallin entre le segment antérieur et postérieur ce qui induit des modifications biochimiques du corps vitré [6] entrainant sa liquéfaction et la survenue d'un décollement postérieur du vitré, augmentant ainsi le risque de déchirures rétiniennes et de décollement de rétine [7-10]. Le délai entre la chirurgie de cataracte et la survenue du DR est variable selon les études. Yoshida a rapporté que 50 % des DR du pseudophaque surviennent la 1ère année après chirurgie, 25 % la 2e année, et 25 % au-delà de 2ans [11]. Dans notre étude, le délai d'apparition était supérieur à 2 ans dans 40 % des cas. La survenue du DR est d'autant plus fréquente s'il y a une forte myopie associée [4]. Plusieurs techniques chirurgicales ont été utilisées pour le traitement du DR du pseudophaque. L'étude de Benson *et al*. réalisée en 1997 sur la technique préférée par les chirurgiens de rétine a noté que 62% préfèrent l'indentation par BS et seulement 7% ont recours à la vitrectomie. Cependant, avec l'évolution des techniques de vitrectomie avec l'utilisation de système grand champ et l'éclairage direct de la rétine périphérique, permet de mieux visualiser les déhiscences périphériques et de mieux apprécier et gérer les tractions vitréo-rétiniennes.

Cependant, les tendances récentes surtout avec l'évolution des techniques de vitrectomie et l'utilisation de système grand champ et l'éclairage direct de la rétine périphérique, optent pour la vitrectomie [2]. Plusieurs études ont démontré les avantages supérieurs de l'abord ab interno par rapport à l'abord ab externo chez les patients pseudophaques [12-14]. La vitrectomie permet de meilleurs résultats anatomiques, moins d'anomalies réfractives, meilleure gestion et traitement des tractions vitré-rétiniennes, l'élimination des cellules ou de l'hémorragie intra vitréenne, meilleure visibilité de la rétine périphérique dont l'examen est souvent gêné par une opacification capsulaire secondaire ,des aberrations optiques générés par les bords de l'implant ou une mauvaise dilatation pupillaire et enfin la possibilité de réaliser une capsulotomie postérieure si nécessaire[13-16]. Dans l'étude réalisée par Campo *et al.* qui a porté sur 225 cas de DRP opéré par vitrectomie en première intention, le succès anatomique était de 88% [17]. Dans notre série, la réapplication anatomique après la première intervention par abord ab intero a été obtenue dans 81,81% des cas. Néanmoins, des complications non négligeables sont possibles, telles qu'une hémorragie intra vitréenne, une déchirure rétinienne iatrogène avec risque de récidive de DR, voire une endophtalmie [18]. Au cours de la chirurgie endo-oculaire, un tamponnement interne par gaz ou par huile de silicone sera réalisé. L'utilisation de gaz pourrait être source d'hypertonie oculaire, de déplacement d'IOL, et de retard de réhabilitation visuelle. Quant à l'utilisation de l'huile de silicone aurait comme inconvénient un passage dans la chambre antérieur [16, 19, 20]. Enfin il faut noter le coût qui est plus élevé lors d'une chirurgie endo-oculaire [21]. L'abord ab externo consiste en un drainage du liquide sous-rétinien avec cryoapplication des déhiscences et indentation épisclérale par bande de silicone. Cette technique est essentiellement indiquée lorsque la ou les déhiscences expliquant la topographie du DR ont été repérées et qui sont aisément indentables sans prolifération vitré-rétinienne avancée.

Les complications de cet abord peuvent être de l'ordre d'une hémorragie sous rétinienne et du décollement choroïdien, qui peuvent être source d'échec thérapeutique compromettant la récupération visuelle postopératoire. Les facteurs d'échec dans notre étude sont liés essentiellement à la présence d'une prolifération vitréo rétinienne avancée, d'une déchirure de novo, et de déchirures non vues à l'examen initial. Enfin, plusieurs études ont comparé les deux techniques chirurgicales, et qui ont noté une légère supériorité de la chirurgie endo-oculaire en terme de résultats anatomiques, cependant le résultat fonctionnel était identique pour les 2 techniques et retrouvé dans plusieurs études [16, 20, 22, 23] notamment l'étude randomisé de Brazitikos [24] qui n'a pas noté de différence significative de l'acuité visuelle finale entre les 2 groupes d'étude. Notre étude rejoint les données de la littérature, certes c'est une étude rétrospective non randomisée avec un faible effectif, cependant nous avons notés des points importants, après un recul moyen de 12mois, nous avons noté un taux de réussite anatomique de 80.76% et de 81.81 % respectivement dans le groupe A et B, reflétant ainsi une efficacité comparable des deux techniques. Ces résultats sont comparables aux autres publications, qui incluaient le même type de décollement de rétine.

## Conclusion

Le décollement de rétine du pseudophaque est une complication grave et relativement fréquente, nécessitant une prise en charge rapide, avec des conditions anatomiques particulières. Certes, avec les avancées technologiques en terme de vitrectomie, la tendance actuelle est la chirurgie endo oculaire, cependant la chirurgie ab-externo garde sa place principalement lors des DR avec des déchirures visibles, bien accessibles à l'indentation sans prolifération vitréorétinienne avancée (PVR A-B).

### Etat des connaissances actuelles sur le sujet

- Tendance actuel à l'abord endo-oculaire du traitement des DDR du pseudophaque;
- Evolution des techniques de vitrectomie et l'utilisation de système grand champ et l'éclairage direct de la rétine périphérique;
- Les causes d'échec restent essentiellement liées à l'importance de la PVR.

### Contribution de notre étude à la connaissance

- La rétinopexie et l'indentation épisclérale reste la technique de référence dans la prise en charge chirurgicale du décollement de rétine (DR) du pseudophaque sans PVR avancée;
- Résultats fonctionnels comparables entre les 2 techniques pour les DR du pseudophaque sans PVR avancée;
- Les causes d'échec dans les deux groupes étaient dues à la présence d'une prolifération vitréorétinienne avancée, d'une déchirure de novo, et de déchirures non vues à l'examen initial.

## Conflits des intérêts

Les auteurs ne déclarent aucun conflit d'intérêts.
